# A novel video game for remote studies of motor adaptation in children

**DOI:** 10.14814/phy2.15764

**Published:** 2023-07-11

**Authors:** Laura A. Malone, Nayo M. Hill, Haley Tripp, Daniel M. Wolpert, Amy J. Bastian

**Affiliations:** ^1^ Kennedy Krieger Institute Baltimore Maryland USA; ^2^ Department of Neurology Johns Hopkins School of Medicine Baltimore Maryland USA; ^3^ Department of Physical Medicine and Rehabilitation Johns Hopkins School of Medicine Baltimore Maryland USA; ^4^ Department of Neuroscience Johns Hopkins School of Medicine Baltimore Maryland USA; ^5^ Mortimer B. Zuckerman Mind Brain Behavior Institute Columbia University New York New York USA; ^6^ Department of Neuroscience Columbia University New York New York USA

**Keywords:** motor adaptation, motor learning, pediatric motor learning, remote administration

## Abstract

Here we designed a motor adaptation video game that could be played remotely (at home) through a web browser. This required the child to adapt to a visuomotor rotation between their hand movement and a ball displayed in the game. The task had several novel features, specifically designed to allow the study of the developmental trajectory of adaptation across a wide range of ages. We test the concurrent validity by comparing children's performance on our remote task to the same task performed in the laboratory. All participants remained engaged and completed the task. We quantified feedforward and feedback control during this task. Feedforward control, a key measure of adaptation, was similar at home and in the laboratory. All children could successfully use feedback control to guide the ball to a target. Traditionally, motor learning studies are performed in a laboratory to obtain high quality kinematic data. However, here we demonstrate concurrent validity of kinematic behavior when conducted at home. Our online platform provides the flexibility and ease of collecting data that will enable future studies with large sample sizes, longitudinal experiments, and the study of children with rare diseases.

## 1 INTRODUCTION

Learning to control movement is an important aspect of human development. Though we are born with some innate motor abilities, learning is required throughout childhood to master motor skills. Motor learning involves many different mechanisms—such as feedback control, error‐based adaptation, and reinforcement learning—that mature at different times during development. This is because the brain structures that are involved in these processes mature at different rates throughout childhood and into adulthood. For example, structural MRI studies have examined when different brain areas achieve peak gray matter volume (a common measure of development). Primary motor and sensory cortices achieve peak gray matter volume around age 5 (Gogtay et al., 2004), the cerebellum around 12–15 years of age (Tiemeier et al., 2010; Wierenga et al., 2014), and different parts of basal ganglia between 7 and 14 years of age (Raznahan et al., 2014; Wierenga et al., 2014).

Currently, there is not a developmental timeline that captures the normal maturation error‐based adaptation. This information would provide accurate estimates of the processes that are typically available to children at different ages and could be used to (i) identify development delays, (ii) understand how damage to the brain affects learning mechanisms, and (iii) identify mechanisms that could be harnessed in rehabilitation.

An error‐based learning process is the driving force behind sensorimotor adaptation. Adaptation is driven by a specific type of error—the difference between where the brain predicted the movement would go and where it actually went. Throughout childhood, the body is constantly changing (e.g., growth spurts and muscle development). Adaptation allows for motor commands from the central nervous system to be updated to these changes in the body or environment. For example, the relation between the visual and actual location of a limb can be altered by wearing prism glasses (Martin et al., 1996). This rotates the visual input so that initial reaching movements are misdirected. Over repeated trials, the reach trajectories adapt to account for the discrepancy caused by the prism. A hallmark of adaptation is the persistence of the newly learned movement when the perturbation is removed (after‐effect).

Studying motor adaptation in childhood is challenging for several reasons. First, the tasks that have been used to measure this process often require people to move in ways that may not be intuitive to children (e.g., absence of hand visual feedback and aiming at invisible targets). Second, some tasks rely on well‐developed cognitive and attentional abilities to follow specific instructions (Holland et al., 2018; Izawa et al., 2008; Therrien et al., 2016), which can be limited in children. Third, methods to test adaptation can require hundreds of repeated movements, which can be monotonous and tiring for children (Holland et al., 2018; Izawa et al., 2008; Scheidt et al., 2000; Therrien et al., 2016, 2018). Fourth, school activities and family commitments can make scheduling appointments to come into the laboratory for experiments difficult for many families.

**FIGURE 1:**
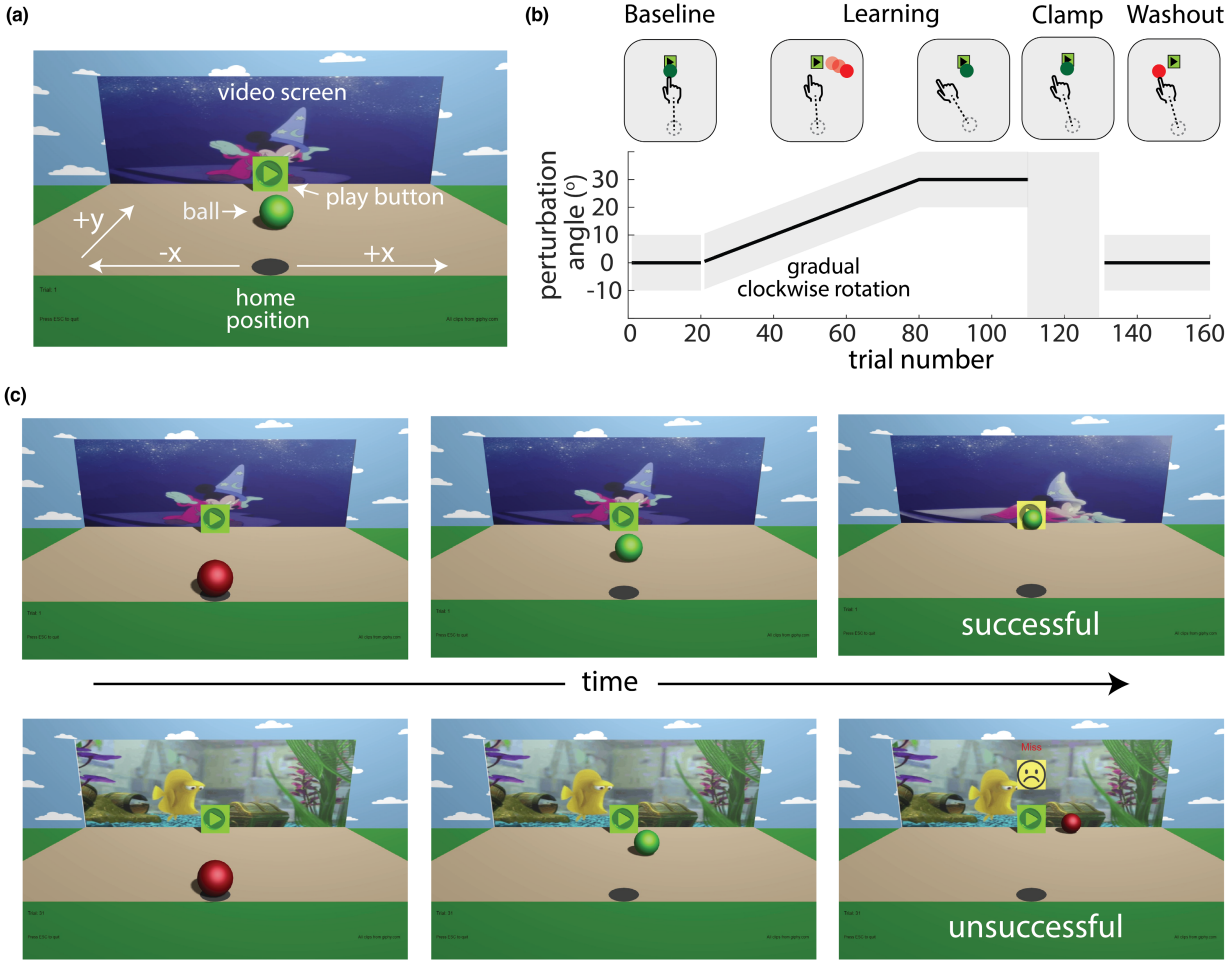
Experimental setup and paradigm. (a) Game environment displayed on a computer screen. Participants clicked on the ball at the home position and then tried to guide the ball to hit the play button. If successful, a brief video clip played on the video screen. Lab participants completed the task in the laboratory using a digital tablet to control the ball. Home participants completed the task remotely using an external mouse. Note that white text and arrows were not displayed to the participants. (b) The task was composed of 160 trials across four blocks: baseline, learning, error clamp, and washout. The plot shows the time course of the visuomotor rotation that was introduced between hand and ball movement. The gray zones show the range of reach angles that would lead to the ball successfully hitting the play button. During baseline and washout, participants received veridical feedback with no perturbation. During learning, a 30‐degree clockwise visual rotation was gradually introduced. During the error clamp, the ball followed the y location of the hand but always moved straight ahead to the target. During this block, participants were always successful. There was a brief rest break between the baseline and learning blocks and between the error clamp and washout blocks. (c) Timeline of a successful (top) and unsuccessful (bottom) trial. White text was not displayed to the participants.

We designed a novel motor learning video game that addresses the issues highlighted above to study the developmental trajectory of sensorimotor adaptation in children. The game is based on a well‐studied error‐based visuomotor rotation task, known to engage adaptation learning mechanisms (Krakauer, 2009). This task has a concrete goal—move a ball to hit a “play” button to start a short video clip (Figure 1a). We utilized gamification techniques to study this task in children (Hamari et al., 2014; Janssen et al., 2017; Listman et al., 2021). Although movement is in two dimensions (the *x*‐*y* position of the mouse controls the *x*‐*y* position of the ball), the ball moves in a three‐dimensional scene with depth cues for a naturalist feel. Task success is indicated by a short engaging Disney cartoon video clip playing on the screen rather than a points score which can have little meaning to younger children. The task is deployed remotely through a web browser to facilitate easy participation of school age children who may have difficulty scheduling time to come to the laboratory enabling data collection from a greater number of children than would be possible with a purely laboratory‐based experiment.

Here, we establish the concurrent validity of task performance in a small subset of children ages 9–16 years old by comparing remote, home play with the traditional administration of motor learning experiments in the laboratory. This age range was selected to span the developmental timeline of the cerebellum, the critical neural structure for adaptation, but include participants are developmentally able to stay focused and on task. Future experiments will study the entire developmental trajectory of children.

## 2 MATERIALS AND METHODS

### 2.1 Subjects

Twenty‐four typically developing children (12 males, 12 females) between 9 and 16 years old (mean ± SD: 11.5 ± 2.5 years) participated in this study, which is a similar sample size to a recent study in adults (Tsay et al., 2021). All participants were recruited via listservs and community postings/events and were screened to rule out neurological, cardiovascular, and developmental conditions. Informed consent was provided by a parent or legal guardian of all participants either in written form (Lab participants) or verbally (Home participants).

### 2.2 Experimental protocol and data collection

All participants completed an error‐based adaptation reaching task in a novel “video game” format. This game was developed using JavaScript to be run from a web browser to collect data from participants of all ages either in the laboratory (Lab) or remotely (Home). The game was hosted on a customized website and data recording and storage were managed through Google Firebase Realtime Database.

To probe adaptation, we gradually introduced a 30‐degree visuomotor rotation between the participant's hand and the ball that they control on the screen. The visuomotor rotation task has been well studied (Blazhenets et al., 2021; Butcher et al., 2017; Canaveral et al., 2017; Codol et al., 2018; Fernandez‐Ruiz et al., 2011; Flannigan et al., 2018; Gaveau et al., 2014; Heuer & Hegele, 2008; Jalali et al., 2017; Krakauer et al., 2005; Lei et al., 2017; Maeda et al., 2017, 2018; Mazzoni & Krakauer, 2006; Neva & Henriques, 2013; Rotella et al., 2013; Tzvi et al., 2022), and performance on this task is similar when conducted remotely or in a laboratory setting in adults (Tsay et al., 2021).

Participants were assigned to one of two groups with the proviso that the groups were matched for age and sex. Participants were naïve to the task. The Lab group participants completed the task using the input device of a Wacom PTH860 Intuos Pro Digital Graphic Drawing Tablet, Large (active area 12.1″ × 8.4″) in the laboratory. They were oriented to the tablet by the experimenter, but then completed the game independently without further experimenter input to minimize any influence of the experimenter on performance. The Home group participants completed the task using their home computer with an input device of a mouse at a time that was convenient for them with no input from the experimenter. For both groups, the game was presented on a screen in front of the participant and the participant controlled the game ball with the input device by moving their arm along the horizontal surface of the table. Visual feedback of hand movement was not restricted for either group. All participants were provided with the same game instructions both in written and spoken formats within the game. All participants were instructed to complete the task all the way through in a single session.

We designed the video game task in a colorful, entertaining, 3D environment to make the experiment fun and engaging for children. The game can be played online in a shortened (https://kidmotorlearning.github.io/AdaptationTask_V8_Demo/index.html) or original length version (https://kidmotorlearning.github.io/AdaptationTask_V8_Full/index.html) with best performance on Google Chrome. At the start of each trial, a still image of a different Disney cartoon video clip is presented on the screen; these were child‐friendly gifs hosted on giphy.com. Participants used their dominant hand to move their finger on the tablet (Lab) or a computer mouse (Home) to control a ball (Figure 1a) to contact the target (a play button) displayed centrally on the screen. Participants initiated a trial by double tapping the tablet or clicking the left mouse button to activate the red game ball (located at 0,0 game units [GU] in the horizontal and vertical screen directions). Note that a game unit is an arbitrary spatial unit in the game environment. Once activated, the ball turned green and was able to be moved towards the target with no additional tapping or clicking. The trial ended once the ball crossed the horizontal plane where the target was located (24 GU forward of the start location), and trajectory data stopped recording. Hitting the target (movement ending within ±10 deg of the target center) caused a short, child‐friendly video clip to play. Missing the target caused a sad face and “Miss” to appear, and the video did not play. Participants in the laboratory made ~6–8 cm movements on the tablet. The distance of the movements with the mouse were dependent on the sensitivity and settings of the home computer and could not be recorded.

The task had four sequential blocks: baseline, learning, error clamp, and washout (Figure 1b). During baseline (20 trials) and washout (30 trials), participants received veridical feedback with no perturbation, meaning the ball position matched the participant's movement. During learning, a 30‐degree clockwise visuomotor rotation was gradually introduced over 60 trials (0.5 deg per trial) and then maintained for a further 30 trials. This rotation created a mismatch between the participant's movement and ball movement on the screen. Clockwise movements were defined as negative angles and counterclockwise positive angles. To be successful, participants had to learn to move the finger/mouse to a location 30 degrees counterclockwise of the target so that the ball would move straight ahead and hit the target (see Figure 1b, second panel in learning).

During the error clamp phase (20 trials), the ball followed the y position of the mouse/tablet but was displayed as moving nearly straight towards the target (with a small, random rotation of −2 to 2 degrees added each trial to mimic natural trial‐to‐trial variation). Therefore, each trial was guaranteed to be successful with little visuomotor error. This phase was used to assess retention of adaptation in absence of error signals that would maintain adaptation (Vaswani et al., 2015; Vaswani & Shadmehr, 2013). There was a brief break for participants between the baseline and learning block and between the error clamp and washout blocks. Participants transitioned seamlessly from the learning block into the error clamp block without any knowledge or instructions that the block had changed.

### 2.3 Data analysis

**FIGURE 2:**
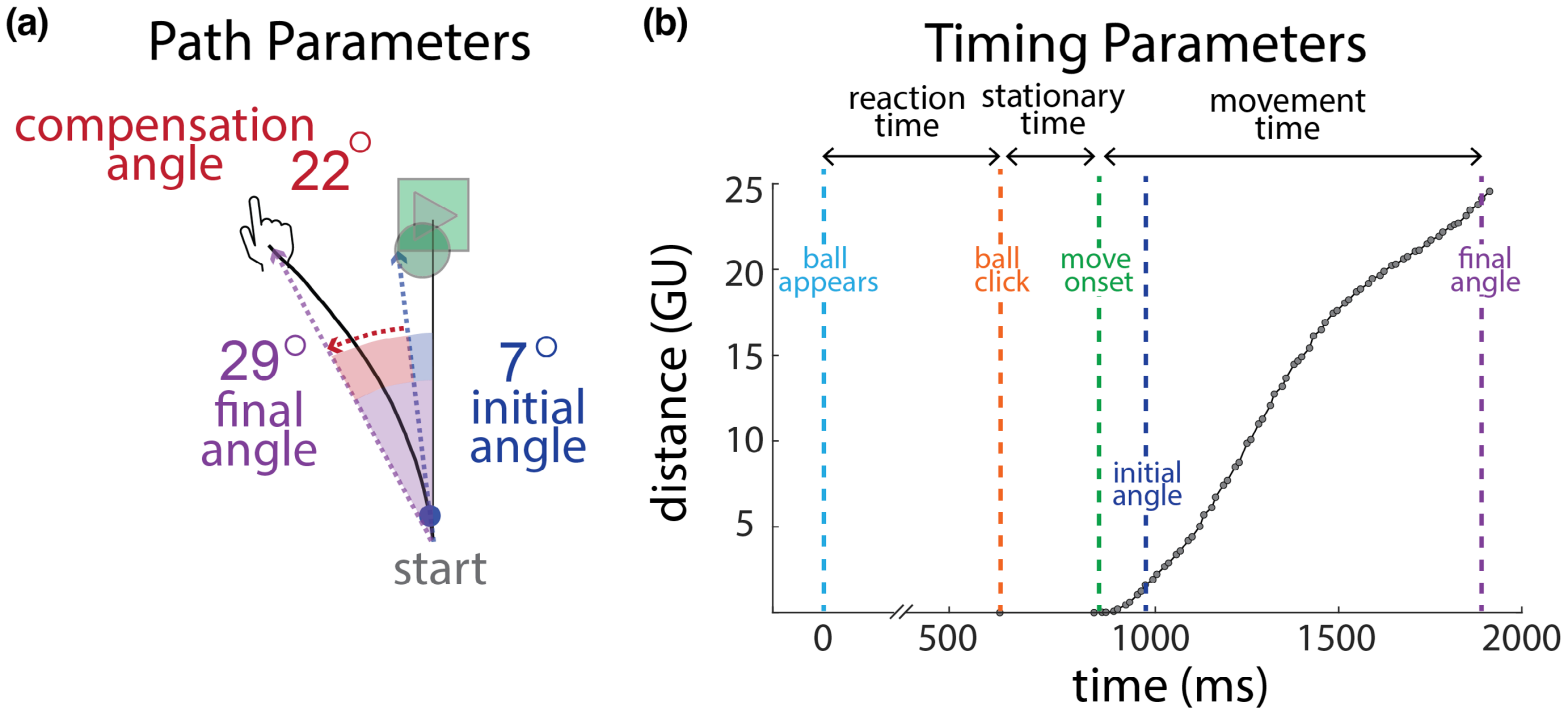
Key performance parameters. (a) The hand path (black line) from a trial late in the learning block (30‐degree perturbation). The *final angle* (purple) is the angle between the line from the start location to the target and the line connecting the hand path's start and end. The *initial angle* (blue) is the angle of the line from the start of the hand path to the point on the path that was 5% of the distance to the target, representing use of feedforward control. The *compensation angle* (red) is the difference between the final and initial angle representing use of online visual feedback. (b) Distance moved plotted as a function of time for a trial from the Home group (points show individual samples of mouse position). Reaction time is defined as time between the ball's appearance and ball click, stationary time is from ball click (orange) to movement onset (green) and movement time is from movement onset (green) to the end of the trajectory (purple, i.e., once the ball crosses the plane of the play button [24 GU]). Note the break in the time axis during the reaction time.

We recorded the movement trajectory of the finger/mouse on every trial. The trajectory data sampled were in the form of change in (delta) mouse/tablet position based on the polling events of the device. When the mouse or finger was stationary, it would not poll and thus not provide data. Sampling rates varied due to the tablet/mouse polling rates but were similar across groups (Lab: 25.5 ± 3.9 Hz and Home: 38.6 ± 6.4 Hz). The “movement angle” is defined as the angle between the (i) finger/mouse to start location direction and (ii) the straight line from the start location to the target (defined as 0 degrees) (solid gray line, Figure 2a).

On each trial, we derived a number of measures to characterize the movement. As has been done previously (Saijo & Gomi, 2010; Schaefer et al., 2009), we used the initial angle (IA) to represent feedforward adaptation (Figure 2a, blue). The initial angle was calculated once participants moved the ball ≥1.2 GU (5%) towards the target. The final angle (the movement angle at the end of the trajectory, Figure 2a, purple) represented overall performance (including whether the trial was a hit or miss). The compensation angle (difference between the final and initial angle, Figure 2a, red) is typically considered a measure of the online feedback used during the movement to steer the ball (Saijo & Gomi, 2010). We also calculated the path length ratio as a measure of path straightness, defined as the ball's path length divided by the distance between the first and last point of the trajectory (Bastian et al., 1996; Chan et al., 2016; Shishov et al., 2017; Struber et al., 2021).

We defined epochs throughout the experiment in order to quantify and compare behavior between the groups (Choi & Bastian, 2007; Coltman & Gribble, 2020; Gidley Larson et al., 2008; Reisman et al., 2005, 2007; Shabbott & Sainburg, 2010). “Baseline” is the last 10 trials of the baseline block. “Learning” is the last 10 trials of the learning block. “Early‐clamp” is the first 3 trials and “late‐clamp” is the last 3 trials of the error clamp block, respectively. “Early‐washout” is the first 3 trials and “end‐washout” is the last 10 trials of the washout block, respectively. In order to adequately capture any transient differences between groups, we average fewer trials in epochs where we expect rapid changes in the behavior (Rossi et al., 2019). The learning amount (LA) was quantified as the difference between the “Learning” and “Baseline” epochs.

Reaction time was defined as the time from trial onset to when the participant clicked the ball. Stationary time was the time from ball click until movement onset (Figure 2b). Movement time was the time from movement onset to the end of the trajectory (final angle on Figure 2b). We did not include stationary time as a part of movement time because children would sometimes click the ball and immediately start moving, whereas other times they would click the ball, wait a period of time, and then initiate movement.

### 2.4 Statistical analysis

Repeated measures ANOVAs (2 × 6) were used to compare the final angle, initial angle, and path length ratio during various epochs of the experiment between the groups. If the assumption of sphericity was violated (Mauchly's test *p* < 0.05), Greenhouse–Geisser corrections were applied. One‐way ANOVAs were used to compare learning amounts (final angle, initial angle, and compensation angle), reaction time, stationary time, time to initial angle, and movement time between groups. Post hoc analyses were performed using Bonferroni test. Outlier trials were removed if the movement time, reaction time, time to initial angle, or path length ratio value was greater than the mean plus three standard deviations (4.5% of trials). SPSS (IBM) was used for all statistical analysis and the alpha level was set at *p* = 0.05. All data are reported as mean ± standard error unless otherwise specified. Standard error was chosen to represent the uncertainty of the true mean of each group and allows for visual comparison of differences of similarities between the groups.

In addition to the frequentist statistics described above, we also used Bayesian analysis. That is, we calculated Bayes Factors (BF) using JASP (Version 0.16.3) [Computer software, https://jasp‐stats.org/download/]. This allowed us to quantify evidence in favor of both the null and the alternative hypotheses (van den Bergh et al., 2020). The Bayes factor represents the relative predictive performance between two hypotheses (typically the null and alternative hypothesis). A Bayes factor of 1 means both hypotheses are equally likely and indistinguishable. The further the Bayes factor deviates from 1, the stronger the evidence for one of the hypotheses. A Bayes factor >3 or <1/3 is considered moderate evidence for the corresponding hypothesis and Bayes factor > 10 or <1/10 is considered strong evidence (Jeffreys, 1961).

## 3 RESULTS

**FIGURE 3:**
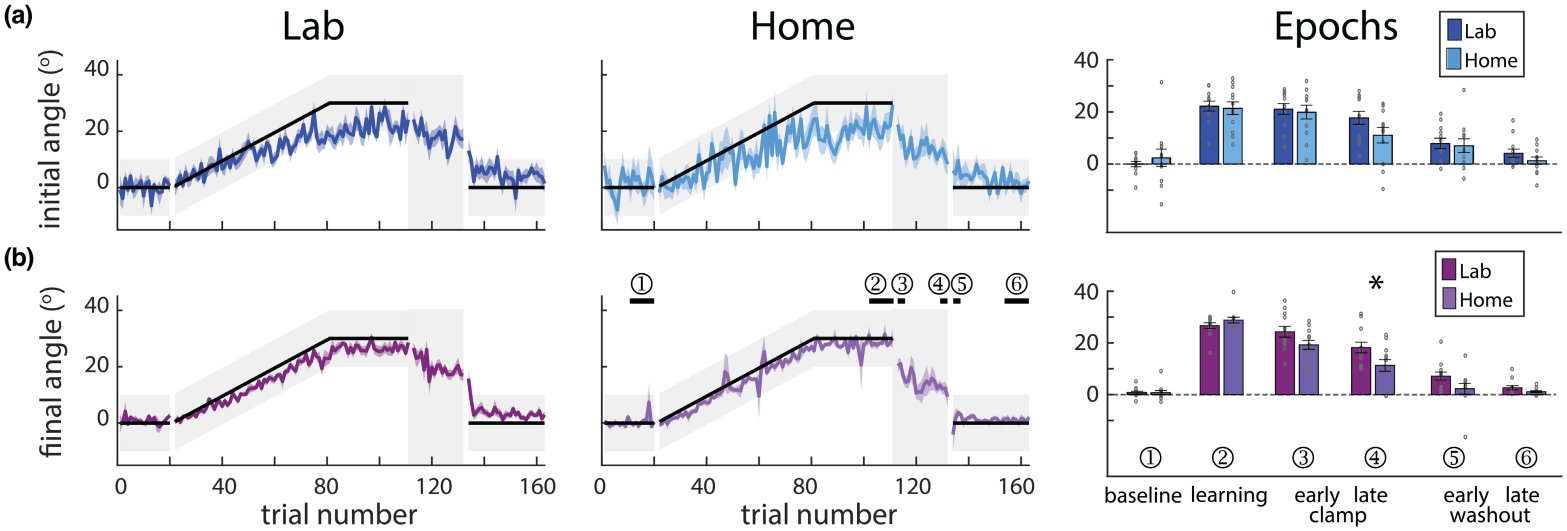
Performance for the Lab and Home groups. (a) The initial angle and (b) final angle over the course of the experiment. Columns 1 and 2 are for Lab and Home groups, respectively, and show mean ± standard error (SE) across participants. Black solid line with gray shaded region demonstrates when participants would be successful at hitting the play button. Right panels show comparisons of epochs (indicated by numbered bars in bottom, middle panel) across the experiment for the two groups (mean ± SE). Individual subjects are shown in gray circles. Statistically significant differences are shown by a * (*p* < 0.05).

All participants were able to complete the video game task without difficulty. Figure 3 shows group data for the initial and final angles. Trial‐by‐trial plots show that both groups change their initial angle (Figure 3a, left and middle panels)—our measure of feedforward adaptation—to account for most of the rotation during adaptation. The initial angle does not return to baseline during the error clamp period (i.e., trials 110–130) indicating retention, and then decays during washout. Figure 3b shows the final angle which includes feedback corrections and determines success. Both groups accounted for the entire rotation and were, on average, within the gray success zone. The final angle did not return to baseline during the clamp but decayed immediately during washout due to feedback corrections (compare initial and final angle during washout).

Importantly, there was no significant difference between Lab and Home initial angle across epochs on the task (Figure 3a, right panel). An ANOVA of initial angle by group and epoch revealed an effect of epoch (*F*_5,110_ = 44.3, *p* < 0.001, Bayes factor > 10^6^), but no group effect (*F*_1,22_ = 0.6, *p* = 0.45, Bayes factor favoring same performance = 2.9) or epoch by group interaction (*F*_5,110_ = 1.25, *p* = 0.29, Bayes factor favoring same performance = 3.3). This demonstrates that participants adapted feedforward control similarly whether completing the Lab or Home tasks, providing validity to the Home data acquisition methodology. Additionally, to compare within subject variability, we measured the variance of the epochs for each subject. There was no significant difference in the variance of the initial angle across epochs between Lab and Home groups (epoch: *F*_5,110_ = 0.864, *p* = 0.51, Bayes factor favoring the same performance 18.9; group: *F*_1,22_ = 1.476, p = 0.24, Bayes factor favoring the same performance = 2.7; epoch by group interaction: *F*_5,110_ = 0.639, *p* = 0.46, Bayes factor favoring the same performance = 87.0).

We saw a small difference between the groups in terms of the final angle across epochs (Figure 3b, right panel). An ANOVA revealed an effect of epoch (*F*_5,110_ = 115.8, *p* < 0.001, Bayes factor = 9.7 × 10^13^), group (*F*_1,22_ = 7.6, *p* = 0.01, Bayes factor = 5), and epoch by group interaction (*F*_5,110_ = 2.6, *p* = 0.047, Bayes factor = 8). On post hoc analysis of the epoch by group interaction, the only epoch that was statistically different was “late‐clamp” (*p* = 0.04). The “early‐clamp” (*p* = 0.08) and “early‐washout” (*p* = 0.07) did not reach significance. Taken together, the small difference that we found in the final angle between the groups are reflective of the Home participants demonstrating less retention. Similar to the initial angle, there was no significant difference in the variance of the final angle across epochs between Lab and Home groups (epoch: *F*_5,110_ = 2.127, *p* = 0.13, Bayes factor favoring the same performance = 2.3; group: *F*_1,22_ = 0.035, *p* = 0.85, Bayes factor favoring the same performance = 4.1; epoch by group interaction: *F*_5,110_ = 1.034, *p* = 0.40, Bayes factor favoring the same performance = 12.7).

**FIGURE 4:**
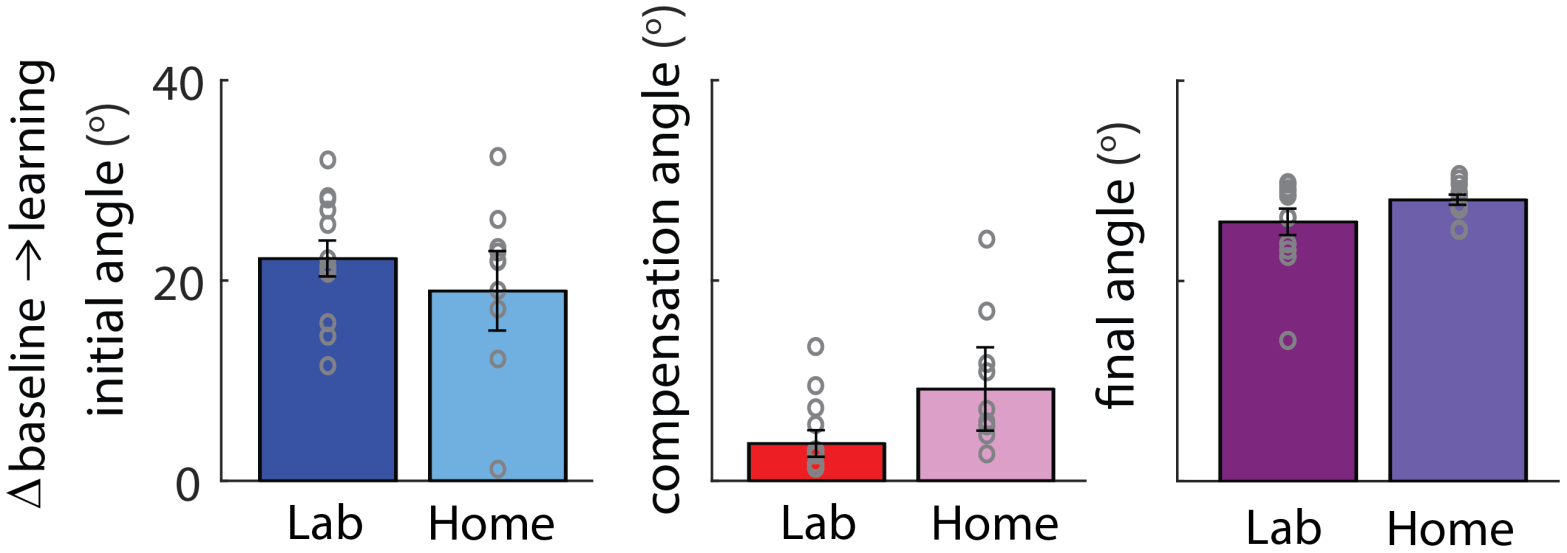
Change in performance from baseline to learning. Plots show change in initial, compensation and final angle for both groups (mean ± SE across participants) from baseline to the end of the learning epoch. Individual subjects are shown in gray circles.

Figure 4 shows the change in initial, compensation, and final angle from baseline to end of learning. We found no significant differences between groups in the initial angle (*F*_1,22_ = 0.6, *p* = 0.47, Bayes factor favoring same performance = 2.2), compensation angle (*F*_1,22_ = 1.55, *p* = 0.23, Bayes factor favoring same performance = 1.5), or final angle (*F*_1,22_ = 2.4, *p* = 0.13, Bayes factor favoring same performance = 1.1). Thus, the Lab and Home groups similarly engaged feedforward adaptation (initial angle) and feedback control (compensation angle) to learn and succeed at the task.

**FIGURE 5:**
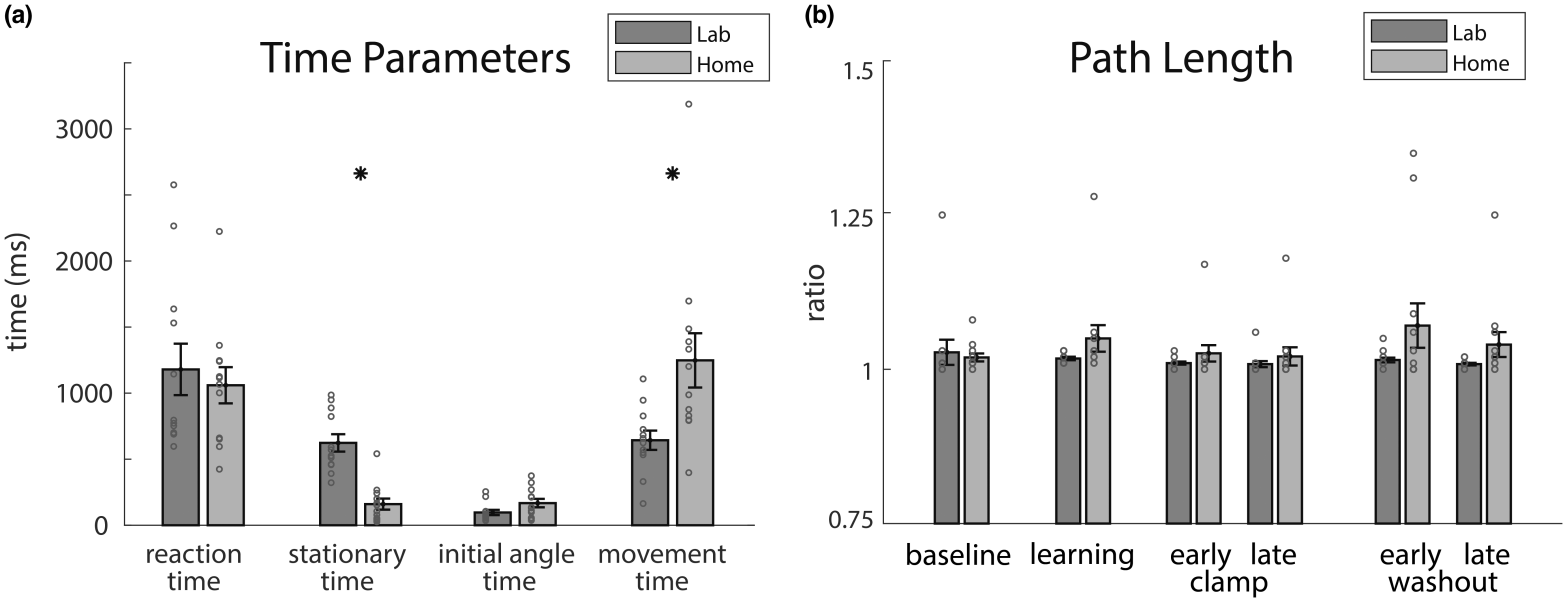
Timing and path length analysis. (a) Timing parameters (mean ± SE across the participants, data were first averaged for all trials within each participant) comparing the Lab and Home groups. (b) Path length ratio (mean ± SE, calculated over all trials within different epochs). Path length ratio was defined as the path length divided by the distance between the first and last point of the path. Individual subjects are shown in gray circles. Statistically significant differences are shown by * (*p* < 0.05).

We then assessed whether other movement parameters differed between the groups. Figure 5a shows the timing parameters measured during the task. Both groups show similar reaction times (*F*_1,22_ = 0.25, *p* = 0.62, Bayes factor favoring same performance = 2.4) and similar initial angle times (*F*_1,22_ = 3.54, *p* = 0.073, Bayes factor favoring same performance = 1.3). The Lab group had longer stationary times (i.e., waiting longer after ball click to start moving) compared to the Home group (623 ± 66 ms vs. 160 ± 42 ms, *F*_1,22_ = 35.3, *p* < 0.001, Bayes factor = 2446). The Lab group also had shorter movement times throughout the task compared to the Home group (643 ± 73 ms vs. 1247 ± 205 ms, *F*_1,22_ = 7.7, *p* = 0.01, Bayes factor = 4.8). We checked to see if the differences in stationary and movement times could be explained by the distance subjects traveled prior to determination of movement onset and found no difference between the two groups (Lab 0.37 ± 0.06 GU vs. Home 0.37 ± 0.13 GU, *F*_1,22_ = 0, *p* = 0.99, Bayes factor favoring same performance = 2.7). We think that Lab participants waited longer to initiate movement because the tablet required a finger tap to select the ball before moving; the Home group could click the mouse and immediately begin moving. Importantly, the time at which we measured the initial angle (IA_time_, 96 ± 20 ms Lab and 167 ± 32 ms Home) was generally less than the visuomotor feedback delay, which is typically 150–180 ms (Miall et al., 1993; Poulton, 1974; Zimmet et al., 2020). Therefore, the initial angle is driven predominantly by feedforward motor mechanisms.

We found that Home participants had longer path length ratio across epochs compared to Lab (Figure 5b), although this did not reach statistical significance (*F*_1,22_ = 3.74, *p* = 0.066, Bayes factor favoring same performance = 1.5) with a repeated measures ANOVA. There was also no epoch (*F*_5,110_ = 0.99, *p* = 0.39, Bayes factor favoring same performance = 14.3) or epoch by group interaction (*F*_5,110_ = 1.18, *p* = 0.32, Bayes factor favoring same performance = 22.6) for the path length ratio.

## 4 DISCUSSION

Although some prior studies have studied adaptation in children (Gómez‐Moya et al., 2016; Ruitenberg et al., 2023; Vasudevan et al., 2011), here we developed a visuomotor adaptation task that can be *administered to children in their homes*. The video game task allowed young children to be successful within a fun and engaging environment. This game enables remote collection of kinematic data from a mouse and replicated findings from the same experiment run in a controlled laboratory setting. Standard clinical and research tests (e.g., Movement Assessment Battery for Children or Bruininks‐Oseretsky Test of Motor Proficiency Ed. 2) assess motor skills or features of motor control (e.g., walking on a balance beam, sorting cards). Here we have designed a task to assess how children *learn* a new movement in a specific way. Although this task is not designed to represent all aspects of sensory‐motor learning during development, it allows for precise testing of one essential motor learning mechanism—adaptation.

This task was designed to overcome the limitations inherent to home data collection. We considered the variability of speed and stability of home internet connections, computer processing speeds, and polling rates and sensitivity of different computer mice. Our task was written in JavaScript which is optimized for web browsers and reduces the processing burden on the computer. We record the polling rates of the mouse to determine whether the participant experienced choppy or delayed game‐play (e.g., if polling goes below 5 Hz). Low polling rates were not an issue in the sample described here, where on average, our Home polling rates were similar or slightly better than the Lab (38.6 vs. 25.5 Hz, respectively). Moreover, we identified specific events within the game, such as the initial and final angle, using spatial thresholds so that we did not have to assume a specific sampling rate.

Additionally, we created a game that would allow engagement and easy participation for children of all ages on their home computer. First, the game had simple instructions that were written and spoken to the child with breaks built in to avoid fatigue. Second, we allowed the player continuous control of the ball so that they could use feedback to correct their movements to be successful. In pilot testing, we found that younger children would quit the game if this was not the case (e.g., if they were asked to aim and release the ball, similar to bowling). Third, we kept the duration of the video clip short (<2.5 s) to make the task quick (~15 min). Fourth, we specifically chose to display a different cartoon video on each trial to help reduce the monotony of completing repetitive trials. Even if children did not get to see the video clip play (i.e., they did not hit the target), they still received engaging stimuli of a new static video to encourage continuation of the task and reduce frustration in very young children. Anecdotally, parents reported that younger children were excited about the different video images/clips and eager to identify the original movies. Desire to see the next image was a way to maintain attention and engagement in the task even in situations of poor performance. Fifth, we provided multiple forms of feedback when children missed (sad face, “Miss” text) to ensure awareness that they did not hit the target. This ensured that children would attribute the video not playing to an error in their movement rather than as an issue with their internet or computer. Sixth, we used the minimum number of trials (160) necessary for participants to adapt and for assessment of retention. Through pilot testing, we found that young children would quit the task due to boredom when the experiment consisted of more trials. Seventh, we utilized the error clamp block in particular to assess retention (as opposed to other ways of assessing retention such as providing no trajectory feedback), because this allowed children to receive positive feedback towards the end of the task when they may be frustrated, tired and at risk of quitting the task early. All of these factors allowed us to collect full data sets easily from the home.

Prior to these design changes, we piloted the task on a group of 28 children across various ages (mean ± SD: 8.3 ± 3.5 years) that were mainly family members of colleagues. This earlier pilot version had a completion rate of 67% with the younger end of the cohort, average age 5.4 years, more likely to quick the task early. In comparison, the completion rate in our current task, incorporating the design components described above, improved to 82% when piloted on 28 children (mean ± SD: 7.4 ± 3 years) unrelated to laboratory members.

Our primary goal was to measure visuomotor adaptation accurately in the home. We show that children adapted similarly whether completing the task with a tablet (Lab) or with a mouse (Home). A hallmark of feedforward adaptation is the presence of a characteristic after‐effect once the perturbation is removed, requiring people to de‐adapt to return to the baseline (Roemmich & Bastian, 2018). In our task, the measure of adaptation was the initial angle which changed to compensate for the perturbation during learning and demonstrated an after‐effect when the perturbation was removed, both in the clamp and washout phase of the experiment. Previous laboratory studies typically measure this based on a timing criteria (Saijo & Gomi, 2010). Because the Home data collection relies on the speed of the family's computer and internet connection, we used a spatial threshold to define the initial angle. The average time to initial angle calculation was 96 ms for Lab and 167 ms for Home players. These latencies are before online feedback would play an appreciable role in correcting the movement (Miall et al., 1993; Poulton, 1974; Zimmet et al., 2020). As such, we think that use of a spatial threshold to define the initial angle is a reasonable representation of the feedforward adaptive motor learning mechanism.

There were also some differences in the behavior of Home participants compared to Lab participants, though these did not impact our measure of adaptation. For example, the final angle, which represents the combination of adaptation and feedback control, showed slightly worse retention in the Home versus Lab group during error clamp and washout blocks. The Home group moved slower compared to Lab players. It may be that the mouse felt less natural than the tablet for children in this age range, which could explain these small differences.

We think that an online, remote administration platform for conducting motor learning experiments offers a new research tool to collect kinematic data from large and diverse samples in an efficient and low‐cost manner. Such approaches have already been used in adults, (Tsay et al., 2021, 2023) but have received less attention in children. Online studies are unlikely to replace laboratory motor learning and motor control studies, especially in circumstances where regular and faster sampling rates, physical perturbation, or kinetics are necessary. However, the flexibility of collecting data remotely allows for more ease in conducting longitudinal or multiple session experiments that require multiple data points from the same participant. This online platform opens the door for studying motor learning and motor control in children throughout the developmental spectrum, in remote or resource limited settings, and in patients with rare diseases.

## AUTHOR CONTRIBUTIONS

Laura Malone: conceptualization, methodology, software, validation, investigation, formal analysis, writing—original draft, visualization. Nayo Hill: methodology, software, writing—review and editing. Haley Tripp: investigation, resources. Daniel Wolpert: methodology, writing—review and editing, supervision, funding acquisition. Amy Bastian: methodology, writing—review and editing, supervision, funding acquisition.

## CONFLICT OF INTEREST STATEMENT

D.M.W is a consultant to CTRL‐Labs Inc., in the Reality Labs Division of Meta. This entity did not support or influence this work. The authors declare no other competing interests.

## ETHICS STATEMENT

These experimental protocols were approved by the Johns Hopkins Institutional Review Board and are consistent with the Helsinki Declaration of 1975 as revised in 2008.

## Data Availability

Data are available upon reasonable request to the corresponding author.

## References

[phy215764-bib-0001] Bastian, A. J. , Martin, T. A. , Keating, J. G. , & Thach, W. T. (1996). Cerebellar ataxia: Abnormal control of interaction torques across multiple joints. Journal of Neurophysiology, 76(1), 492–509. 10.1152/jn.1996.76.1.492 8836239

[phy215764-bib-0002] Blazhenets, G. , Kurz, A. , Frings, L. , Leukel, C. , & Meyer, P. T. (2021). Brain activation patterns during visuomotor adaptation in motor experts and novices: An FDG PET study with unrestricted movements. Journal of Neuroscience Methods, 350, 109061. 10.1016/j.jneumeth.2020.109061 33370559

[phy215764-bib-0003] Butcher, P. A. , Ivry, R. B. , Kuo, S. H. , Rydz, D. , Krakauer, J. W. , & Taylor, J. A. (2017). The cerebellum does more than sensory prediction error‐based learning in sensorimotor adaptation tasks. Journal of Neurophysiology, 118(3), 1622–1636. 10.1152/jn.00451.2017 28637818 PMC5596119

[phy215764-bib-0004] Canaveral, C. A. , Danion, F. , Berrigan, F. , & Bernier, P. M. (2017). Variance in exposed perturbations impairs retention of visuomotor adaptation. Journal of Neurophysiology, 118(5), 2745–2754. 10.1152/jn.00416.2017 28814633 PMC5675903

[phy215764-bib-0005] Chan, I. H. L. , Fong, K. N. K. , Chan, D. Y. L. , Wang, A. Q. , Cheng, E. K. , Chau, P. H. , Chow, K. K. , & Cheung, H. K. (2016). Effects of arm weight support training to promote recovery of upper limb function for subacute patients after stroke with different levels of arm impairments. BioMed Research International, 2016, 9346374. 10.1155/2016/9346374 27517053 PMC4969527

[phy215764-bib-0006] Choi, J. T. , & Bastian, A. J. (2007). Adaptation reveals independent control networks for human walking. Nature Neuroscience, 10(8), 1055–1062. 10.1038/nn1930 17603479

[phy215764-bib-0007] Codol, O. , Holland, P. J. , & Galea, J. M. (2018). The relationship between reinforcement and explicit control during visuomotor adaptation. Scientific Reports, 8(1), 9121. 10.1038/s41598-018-27378-1 29904096 PMC6002524

[phy215764-bib-0008] Coltman, S. K. , & Gribble, P. L. (2020). Time course of changes in the long‐latency feedback response parallels the fast process of short‐term motor adaptation. Journal of Neurophysiology, 124(2), 388–399. 10.1152/jn.00286.2020 32639925 PMC7500369

[phy215764-bib-0009] Fernandez‐Ruiz, J. , Wong, W. , Armstrong, I. T. , & Flanagan, J. R. (2011). Relation between reaction time and reach errors during visuomotor adaptation. Behavioural Brain Research, 219(1), 8–14. 10.1016/j.bbr.2010.11.060 21138745

[phy215764-bib-0010] Flannigan, J. C. , Posthuma, R. J. , Lombardo, J. N. , Murray, C. , & Cressman, E. K. (2018). Adaptation to proprioceptive targets following visuomotor adaptation. Experimental Brain Research, 236(2), 419–432. 10.1007/s00221-017-5141-y 29209829

[phy215764-bib-0011] Gaveau, V. , Prablanc, C. , Laurent, D. , Rossetti, Y. , & Priot, A. E. (2014). Visuomotor adaptation needs a validation of prediction error by feedback error. Frontiers in Human Neuroscience, 8, 880. 10.3389/fnhum.2014.00880 25408644 PMC4219430

[phy215764-bib-0012] Gidley Larson, J. C. , Bastian, A. J. , Donchin, O. , Shadmehr, R. , & Mostofsky, S. H. (2008). Acquisition of internal models of motor tasks in children with autism. Brain, 131(11), 2894–2903. 10.1093/brain/awn226 18819989 PMC2577807

[phy215764-bib-0013] Gogtay, N. , Giedd, J. N. , Lusk, L. , Hayashi, K. M. , Greenstein, D. , Vaituzis, A. C. , Nugent, T. F., III , Herman, D. H. , Clasen, L. S. , Toga, A. W. , Rapoport, J. L. , & Thompson, P. M. (2004). Dynamic mapping of human cortical development during childhood through early adulthood. Proceedings of the National Academy of Sciences of the United States of America, 101(21), 8174–8179. 10.1073/pnas.0402680101 15148381 PMC419576

[phy215764-bib-0014] Gómez‐Moya, R. , Díaz, R. , & Fernandez‐Ruiz, J. (2016). Different visuomotor processes maturation rates in children support dual visuomotor learning systems. Human Movement Science, 46, 221–228. 10.1016/j.humov.2016.01.011 26802974

[phy215764-bib-0015] Hamari, J. , Koivisto, J. , & Sarsa, H. (2014). Does gamification work?—A literature review of empirical studies on gamification (pp. 3025–3034). 2014 47th Hawaii International Conference on System Sciences. 10.1109/HICSS.2014.377

[phy215764-bib-0016] Heuer, H. , & Hegele, M. (2008). Adaptation to visuomotor rotations in younger and older adults. Psychology and Aging, 23(1), 190–202. 10.1037/0882-7974.23.1.190 18361666

[phy215764-bib-0017] Holland, P. , Codol, O. , & Galea, J. M. (2018). Contribution of explicit processes to reinforcement‐based motor learning. Journal of Neurophysiology, 119(6), 2241–2255. 10.1152/jn.00901.2017 29537918 PMC6032115

[phy215764-bib-0018] Izawa, J. , Rane, T. , Donchin, O. , & Shadmehr, R. (2008). Motor adaptation as a process of Reoptimization. The Journal of Neuroscience, 28(11), 2883–2891. 10.1523/JNEUROSCI.5359-07.2008 18337419 PMC2752329

[phy215764-bib-0019] Jalali, R. , Miall, R. C. , & Galea, J. M. (2017). No consistent effect of cerebellar transcranial direct current stimulation on visuomotor adaptation. Journal of Neurophysiology, 118(2), 655–665. 10.1152/jn.00896.2016 28298304 PMC5539446

[phy215764-bib-0020] Janssen, J. , Verschuren, O. , Renger, W. J. , Ermers, J. , Ketelaar, M. , & van Ee, R. (2017). Gamification in physical therapy: More than using games. Pediatric Physical Therapy, 29(1), 95–99. 10.1097/PEP.0000000000000326 27984481

[phy215764-bib-0021] Jeffreys, H. (1961). The theory of probability. Third.

[phy215764-bib-0022] Krakauer, J. W. (2009). Motor learning and consolidation: The case of visuomotor rotation. Advances in Experimental Medicine and Biology, 629, 405–421. 10.1007/978-0-387-77064-2_21 19227512 PMC2672910

[phy215764-bib-0023] Krakauer, J. W. , Ghez, C. , & Ghilardi, M. F. (2005). Adaptation to visuomotor transformations: Consolidation, interference, and forgetting. The Journal of Neuroscience, 25(2), 473–478. 10.1523/JNEUROSCI.4218-04.2005 15647491 PMC6725486

[phy215764-bib-0024] Lei, Y. , Bao, S. , Perez, M. A. , & Wang, J. (2017). Enhancing generalization of visuomotor adaptation by inducing use‐dependent learning. Neuroscience, 366, 184–195. 10.1016/j.neuroscience.2017.10.004 29031601

[phy215764-bib-0025] Listman, J. B. , Tsay, J. S. , Kim, H. E. , Mackey, W. E. , & Heeger, D. J. (2021). Long‐term motor learning in the “wild” with high volume video game data. Frontiers in Human Neuroscience., 15, 15. 10.3389/fnhum.2021.777779 PMC8720934 34987368

[phy215764-bib-0026] Maeda, R. S. , McGee, S. E. , & Marigold, D. S. (2017). Consolidation of visuomotor adaptation memory with consistent and noisy environments. Journal of Neurophysiology, 117(1), 316–326. 10.1152/jn.00178.2016 27784800 PMC5225953

[phy215764-bib-0027] Maeda, R. S. , McGee, S. E. , & Marigold, D. S. (2018). Long‐term retention and reconsolidation of a visuomotor memory. Neurobiology of Learning and Memory, 155, 313–321. 10.1016/j.nlm.2018.08.020 30172955

[phy215764-bib-0028] Martin, T. A. , Keating, J. G. , Goodkin, H. P. , Bastian, A. J. , & Thach, W. T. (1996). Throwing while looking through prisms. I. Focal olivocerebellar lesions impair adaptation. Brain, 119(Pt 4), 1183–1198. 10.1093/brain/119.4.1183 8813282

[phy215764-bib-0029] Mazzoni, P. , & Krakauer, J. W. (2006). An implicit plan overrides an explicit strategy during visuomotor adaptation. The Journal of Neuroscience, 26(14), 3642–3645. 10.1523/JNEUROSCI.5317-05.2006 16597717 PMC6674132

[phy215764-bib-0030] Miall, R. C. , Weir, D. J. , Wolpert, D. M. , & Stein, J. F. (1993). Is the cerebellum a smith predictor? Journal of Motor Behavior, 25(3), 203–216. 10.1080/00222895.1993.9942050 12581990

[phy215764-bib-0031] Neva, J. L. , & Henriques, D. Y. P. (2013). Visuomotor adaptation and generalization with repeated and varied training. Experimental Brain Research, 226(3), 363–372. 10.1007/s00221-013-3444-1 23455723

[phy215764-bib-0032] Poulton, E. C. (1974). Tracking skill and manual control. Academic.

[phy215764-bib-0033] Raznahan, A. , Shaw, P. W. , Lerch, J. P. , Clasen, L. S. , Greenstein, D. , Berman, R. , Pipitone, J. , Chakravarty, M. M. , & Giedd, J. N. (2014). Longitudinal four‐dimensional mapping of subcortical anatomy in human development. Proceedings of the National Academy of Sciences of the United States of America, 111(4), 1592–1597. 10.1073/pnas.1316911111 24474784 PMC3910572

[phy215764-bib-0034] Reisman, D. S. , Block, H. J. , & Bastian, A. J. (2005). Interlimb coordination during locomotion: What can be adapted and stored? Journal of Neurophysiology, 94(4), 2403–2415. 10.1152/jn.00089.2005 15958603

[phy215764-bib-0035] Reisman, D. S. , Wityk, R. , Silver, K. , & Bastian, A. J. (2007). Locomotor adaptation on a split‐belt treadmill can improve walking symmetry post‐stroke. Brain, 130(Pt 7), 1861–1872. 10.1093/brain/awm035 17405765 PMC2977955

[phy215764-bib-0036] Roemmich, R. T. , & Bastian, A. J. (2018). Closing the loop: From motor neuroscience to neurorehabilitation. Annual Review of Neuroscience, 41(1), 415–429. 10.1146/annurev-neuro-080317-062245 29709206

[phy215764-bib-0037] Rossi, C. , Chau, C. W. , Leech, K. A. , Statton, M. A. , Gonzalez, A. J. , & Bastian, A. J. (2019). The capacity to learn new motor and perceptual calibrations develops concurrently in childhood. Scientific Reports, 9, 9322. 10.1038/s41598-019-45074-6 31249379 PMC6597729

[phy215764-bib-0038] Rotella, M. F. , Koehler, M. , Nisky, I. , Bastian, A. J. , & Okamura, A. M. (2013). Adaptation to visuomotor rotation in isometric reaching is similar to movement adaptation. IEEE International Conference on Rehabilitation Robotics, 2013, 6650431. 10.1109/ICORR.2013.6650431 24187249

[phy215764-bib-0039] Ruitenberg, M. F. L. , Koppelmans, V. , Seidler, R. D. , & Schomaker, J. (2023). Developmental and age differences in visuomotor adaptation across the lifespan. Psychological Research, 9. 10.1007/s00426-022-01784-7 PMC10366290 36617621

[phy215764-bib-0040] Saijo, N. , & Gomi, H. (2010). Multiple motor learning strategies in visuomotor rotation. PLoS One, 5(2), e9399. 10.1371/journal.pone.0009399 20195373 PMC2827554

[phy215764-bib-0041] Schaefer, S. Y. , Haaland, K. Y. , & Sainburg, R. L. (2009). Dissociation of initial trajectory and final position errors during visuomotor adaptation following unilateral stroke. Brain Research, 1298, 78–91. 10.1016/j.brainres.2009.08.063 19728993 PMC3151492

[phy215764-bib-0042] Scheidt, R. A. , Reinkensmeyer, D. J. , Conditt, M. A. , Rymer, W. Z. , & Mussa‐Ivaldi, F. A. (2000). Persistence of motor adaptation during constrained, multi‐joint, arm movements. Journal of Neurophysiology, 84(2), 853–862. 10.1152/jn.2000.84.2.853 10938312

[phy215764-bib-0043] Shabbott, B. A. , & Sainburg, R. L. (2010). Learning a visuomotor rotation: Simultaneous visual and proprioceptive information is crucial for visuomotor remapping. Experimental Brain Research, 203(1), 75–87. 10.1007/s00221-010-2209-3 20237773 PMC3702748

[phy215764-bib-0044] Shishov, N. , Melzer, I. , & Bar‐Haim, S. (2017). Parameters and measures in assessment of motor learning in neurorehabilitation; a systematic review of the literature. Frontiers in Human Neuroscience, 11, 82. 10.3389/fnhum.2017.00082 28286474 PMC5324661

[phy215764-bib-0045] Struber, L. , Baumont, M. , Barraud, P. A. , Nougier, V. , & Cignetti, F. (2021). Brain oscillatory correlates of visuomotor adaptive learning. NeuroImage, 245, 118645. 10.1016/j.neuroimage.2021.118645 34687861

[phy215764-bib-0046] Therrien, A. S. , Wolpert, D. M. , & Bastian, A. J. (2016). Effective reinforcement learning following cerebellar damage requires a balance between exploration and motor noise. Brain, 139(1), 101–114. 10.1093/brain/awv329 26626368 PMC4949390

[phy215764-bib-0047] Therrien, A. S. , Wolpert, D. M. , & Bastian, A. J. (2018). Increasing motor noise impairs reinforcement learning in healthy individuals. eNeuro, 5(3), ENEURO.0050–ENEURO18.2018. 10.1523/ENEURO.0050-18.2018 30105298 PMC6088368

[phy215764-bib-0048] Tiemeier, H. , Lenroot, R. K. , Greenstein, D. K. , Tran, L. , Pierson, R. , & Giedd, J. N. (2010). Cerebellum development during childhood and adolescence: A longitudinal morphometric MRI study. NeuroImage, 49(1), 63–70. 10.1016/j.neuroimage.2009.08.016 19683586 PMC2775156

[phy215764-bib-0049] Tsay, J. S. , Asmerian, H. , Germine, L. T. , Wilmer, J. , Ivry, R. B. , & Nakayama, K. (2023). Predictors of sensorimotor adaption: Insights from over 100,000 reaches. bioRxiv. 10.1101/2023.01.18.524634

[phy215764-bib-0050] Tsay, J. S. , Lee, A. S. , Ivry, R. B. , & Avraham, G. (2021). Moving outside the lab: The viability of conducting sensorimotor learning studies online. bioRxiv. 10.1101/2021.01.30.181370

[phy215764-bib-0051] Tzvi, E. , Loens, S. , & Donchin, O. (2022). Mini‐review: The role of the cerebellum in visuomotor adaptation. Cerebellum, 21(2), 306–313. 10.1007/s12311-021-01281-4 34080132 PMC8993777

[phy215764-bib-0052] van den Bergh, D. , van Doorn, J. , Marsman, M. , Draws, T. , van Kesteren, E. J. , Derks, K. , Dablander, F. , Gronau, Q. F. , Kucharský, Š. , Gupta, A. R. K. N. , Sarafoglou, A. , Voelkel, J. G. , Stefan, A. , Ly, A. , Hinne, M. , Matzke, D. , & Wagenmakers, E. J. (2020). A tutorial on conducting and interpreting a Bayesian ANOVA in JASP. L'Année Psychologique, 120(1), 73–96. 10.3917/anpsy1.201.0073

[phy215764-bib-0053] Vasudevan, E. V. L. , Torres‐Oviedo, G. , Morton, S. M. , Yang, J. F. , & Bastian, A. J. (2011). Younger is not always better: Development of locomotor adaptation from childhood to adulthood. The Journal of Neuroscience, 31(8), 3055–3065. 10.1523/JNEUROSCI.5781-10.2011 21414926 PMC3084584

[phy215764-bib-0054] Vaswani, P. A. , & Shadmehr, R. (2013). Decay of motor memories in the absence of error. The Journal of Neuroscience, 33(18), 7700–7709. 10.1523/JNEUROSCI.0124-13.2013 23637163 PMC3712872

[phy215764-bib-0055] Vaswani, P. A. , Shmuelof, L. , Haith, A. M. , Delnicki, R. J. , Huang, V. S. , Mazzoni, P. , Shadmehr, R. , & Krakauer, J. W. (2015). Persistent residual errors in motor adaptation tasks: Reversion to baseline and exploratory escape. The Journal of Neuroscience, 35(17), 6969–6977. 10.1523/JNEUROSCI.2656-14.2015 25926471 PMC4412906

[phy215764-bib-0056] Wierenga, L. , Langen, M. , Ambrosino, S. , van Dijk, S. , Oranje, B. , & Durston, S. (2014). Typical development of basal ganglia, hippocampus, amygdala and cerebellum from age 7 to 24. NeuroImage, 96, 67–72. 10.1016/j.neuroimage.2014.03.072 24705201

[phy215764-bib-0057] Zimmet, A. M. , Cao, D. , Bastian, A. J. , & Cowan, N. J. (2020). Cerebellar patients have intact feedback control that can be leveraged to improve reaching. eLife, 9, 9. 10.7554/eLife.53246 PMC7577735 33025903

